# Evaluation of the nurse-assisted eHealth intervention ‘eHealth@Hospital-2-Home’ on self-care by patients with heart failure and colorectal cancer post-hospital discharge: protocol for a randomised controlled trial

**DOI:** 10.1186/s12913-023-10508-5

**Published:** 2024-01-04

**Authors:** Marianne Storm, Ingvild Margreta Morken, Rosalynn C. Austin, Oda Nordfonn, Hege Bjøkne Wathne, Kristin Hjorthaug Urstad, Bjørg Karlsen, Ingvild Dalen, Kari Hanne Gjeilo, Alison Richardson, Glyn Elwyn, Edvin Bru, Jon Arne Søreide, Hartwig Kørner, Rune Mo, Anna Strömberg, Hilde Lurås, Anne Marie Lunde Husebø

**Affiliations:** 1 Department of Public Health, Faculty of Health Sciences, University of Stavanger, 4036 Stavanger, Norway; 2 Faculty of Health Sciences and Social Care, Molde University College, Molde, Norway; 3 Research Group of Nursing and Health Sciences, Research Department, Stavanger University Hospital, Stavanger, Norway; 4 Department of Quality and Health Technologies, Faculty of Health Sciences, University of Stavanger, Stavanger, Norway; 5 Department of Cardiology, Portsmouth Hospitals University NHS Trust, Portsmouth, UK; 6 National Institute of Health and Care Research (NIHR) Applied Research Collaborative (ARC) Wessex, Southampton, SO17 1BJ UK; 7 Department of Health and Caring Science, Western Norway University of Applied Science, Stord, Norway; 8 Section of Biostatistics, Department of Research, Stavanger University Hospital, Stavanger, Norway; 9 Department of Public Health and Nursing, Faculty of Medicine, and Health Sciences, NTNU – Norwegian University of Science and Technology, Trondheim, Norway; 10 Department of Cardiology, St. Olav’s Hospital, Trondheim University Hospital, Trondheim, Norway; 11 University Hospital Southampton NHS Foundation Trust, Southampton General Hospital, Mailpoint 11, Clinical Academic Facility (Room AA102), South Academic Block, Tremona Road, Southampton, SO16 6YD UK; 12 The Dartmouth Institute for Health Policy and Clinical Practice at the Geisel School of Medicine at Dartmouth College, Hanover, NH USA; 13 Centre for Learning Environment, University of Stavanger, Stavanger, Norway; 14 Department of Gastrointestinal Surgery, Stavanger University Hospital, Stavanger, Norway; 15 Department of Clinical Medicine, University of Bergen, Bergen, Norway; 16 Department of Cardiology, St. Olav’s Hospital, and Trondheim University Hospital, Trondheim, Norway; 17 Department of Circulation and Medical Imaging, Faculty of Medicine, and Health Sciences, NTNU – Norwegian University of Science and Technology, Trondheim, Norway; 18 Department of Health, Medicine and Caring Sciences, Linköping University, Linköping, Sweden; 19 Department of Cardiology, Linköping University, Linköping, Sweden; 20 Faculty of Health Studies, VID Specialized University, Oslo, Norway; 21 Avdeling for Helsetjenesteforskning (HØKH), Akershus University Hospital, Lørenskog, Norway; 22 Institute of Clinical Medicine, University of Oslo, Oslo, Norway

**Keywords:** Heart failure, Colorectal cancer, Self-efficacy, eHealth, Randomised controlled trial, Hospital discharge, Protocol

## Abstract

**Background:**

Patients with heart failure (HF) and colorectal cancer (CRC) are prone to comorbidity, a high rate of readmission, and complex healthcare needs. Self-care for people with HF and CRC after hospitalisation can be challenging, and patients may leave the hospital unprepared to self-manage their disease at home. eHealth solutions may be a beneficial tool to engage patients in self-care.

**Methods:**

A randomised controlled trial with an embedded evaluation of intervention engagement and cost-effectiveness will be conducted to investigate the effect of eHealth intervention after hospital discharge on the self-efficacy of self-care. Eligible patients with HF or CRC will be recruited before discharge from two Norwegian university hospitals. The intervention group will use a nurse-assisted intervention—eHealth@Hospital-2-Home—for six weeks. The intervention includes remote monitoring of vital signs; patients’ self-reports of symptoms, health and well-being; secure messaging between patients and hospital-based nurse navigators; and access to specific HF and CRC health-related information. The control group will receive routine care. Data collection will take place before the intervention (baseline), at the end of the intervention (Post-1), and at six months (Post-2). The primary outcome will be self-efficacy in self-care. The secondary outcomes will include measures of burden of treatment, health-related quality of life and 30- and 90-day readmissions. Sub-study analyses are planned in the HF patient population with primary outcomes of self-care behaviour and secondary outcomes of medication adherence, and readmission at 30 days, 90 days and 6 months. Patients’ and nurse navigators’ engagement and experiences with the eHealth intervention and cost-effectiveness will be investigated. Data will be analysed according to intention-to-treat principles. Qualitative data will be analysed using thematic analysis.

**Discussion:**

This protocol will examine the effects of the eHealth@ Hospital-2-Home intervention on self-care in two prevalent patient groups, HF and CRC. It will allow the exploration of a generic framework for an eHealth intervention after hospital discharge, which could be adapted to other patient groups, upscaled, and implemented into clinical practice.

**Trial registration:**

Clinical trials.gov (ID 301472).

## Background

Worldwide healthcare systems are increasingly relying on patients to manage their treatment and illness through self-care due to the rising rates and pressures created by chronic illness [1, 2]. In Norway and globally, cancer and cardiovascular conditions are illnesses with considerable burdens for patients and healthcare costs [3]. In 2020, approximately 15,000 Norwegians were diagnosed with heart failure (HF) [4] with the prevalence of known HF being 1–2% of the global population [5]. In 2020, nearly 4,500 Norwegians were diagnosed with colorectal cancer (CRC) with the prevalence increasing by 1.8% per year in Norway between 2012 and 2016 [6]. Both conditions are prone to comorbidity and complex healthcare needs, including high readmission rates [5, 7–9].

While the two illnesses are different in terms of diagnosis, treatment, prognosis and trajectory [10, 11], HF and CRC have similar needs for follow-up care and the development of patients’ self-care skills [12, 13]. Individuals with either HF or CRC have challenges with unmet health needs and limited access to healthcare professionals, particularly after hospitalisation [14–16]. Between hospital admissions and specialist appointments, patients must self-care at home [17]. Patients also often receive limited support with the transition from hospital to home [18]. Inadequate follow-up of HF and CRC patients after hospitalisation can potentially result in unsafe and anxious patients [16, 19], which may lead to poor adherence to medical treatment and self-care tasks [20], resulting in readmission [21] and low quality of life [22]. Unsupportive healthcare service pathways lead to a higher burden of treatment [23].

The burden of treatment is the delicate balance between a patient’s capacity (capability and resources) and the workload (tasks required by illness management and treatments) [24]. If that balance tips so the workload exceeds the patient’s capacity, patients experience poorer outcomes, health-related quality of life (HRQoL) [25] and self-care [24]. Self-care refers to ‘the ability of the patient to deal with a chronic illness including symptoms, treatment, physical and social consequences, and lifestyle changes’ [26]. It is a complex construct with multiple processes and influencing factors [27]. Self-efficacy is key to influencing patient decisions around self-care behaviours [27]. It refers to an individual’s confidence in their capacity to act in the ways necessary to reach specific goals [28] and is linked to self-management [1].

eHealth solutions may provide a way to support self-care in illnesses such as HF and CRC. eHealth is both the delivery of health services and information [29] as well as the conceptualisation of user interactions with the technology (e.g., monitoring of vital signs, communication with healthcare providers and utilisation of data to restore health) [30]. Patients who use eHealth may be more motivated to engage in self-care behaviours [31, 32]. eHealth-based self-care interventions have been found to improve confidence in self-care of cancer patients and coronary heart disease patients [33, 34]. Within HF, a review of eHealth follow-up programmes has reported significantly increased HRQoL, but the impact on readmissions and self-care is less clear [35]. In cancer survivors, Chan et al. [36] reported that telemedicine significantly improved HRQoL and symptom burden. A cost-effectiveness review suggested that nurse-assisted follow-up is a promising method for reducing costs (compared with rehospitalisation) without endangering patient outcomes, following cancer treatment [37].

Research on how eHealth can best support patients with HF and CRC during the period after hospital discharge is sparse [36, 38]. Therefore, a nurse-assisted eHealth intervention eHealth@Hospital-2-Home was developed and tested based on a body of research in HF and CRC patients [15, 35, 39, 40]. Existing software and application technology—the Dignio Connected Care [41]—was selected as the platform for the intervention. The eHealth intervention includes:

- monitoring of vital signs using connected technology (heart rate, blood pressure, pulse oximetry, temperature, weight)
- patient self-reporting of symptoms, health and well-being
- secure messaging between patients and hospital-based nurse navigators when patients’ vital signs exceed the acceptable range
- access to HF and CRC health-related educational information.

This paper presents the protocol for a randomised controlled trial (RCT) evaluating a complex intervention [42] to study the effects of the eHealth@Hospital-2-Home intervention on the primary outcome of self-efficacy in self-care in HF and CRC patients. The protocol was informed by the preliminary results from a feasibility study with the two patient groups [43] and follows the recommendations for protocol items for a clinical trial (SPIRIT) checklist [44] and the CONSORT checklist for reporting items of a randomised trial [45].

The aims of the RCT are as follows.

Primary aims:

- 1) To evaluate the immediate (Post-1: 6 weeks) and extended effects (Post-2: 6 months) of an eHealth intervention after hospital discharge to improve patients’ self-efficacy to self-manage chronic illness. We hypothesise that participants in the intervention group will report higher levels of self-efficacy at six weeks after hospital discharge and at six months after baseline compared with the control group.

Secondary aims:

- 2) To investigate the effects of the intervention on the secondary outcomes of the burden of treatment, HRQoL, perceived collaboration and social support from healthcare personnel, healthcare utilisation and 30- and 90-day readmission rates in the two patient groups.
- 3) HF sub-study: To investigate the effects of the intervention on self-care behaviour, medication compliance and 6- and 12-month readmissions in participants with HF only. We hypothesise that the HF intervention group will report higher levels of self-care behaviours, better medication compliance and reduced 6- and 12-month hospital readmissions compared with the HF control group.

Explorative aim:

- 4) To evaluate patients’ and nurse navigators’ engagement and experiences with the eHealth intervention and estimate cost-effectiveness. Evaluation will use mixed methods [42].

**Fig. 1:**
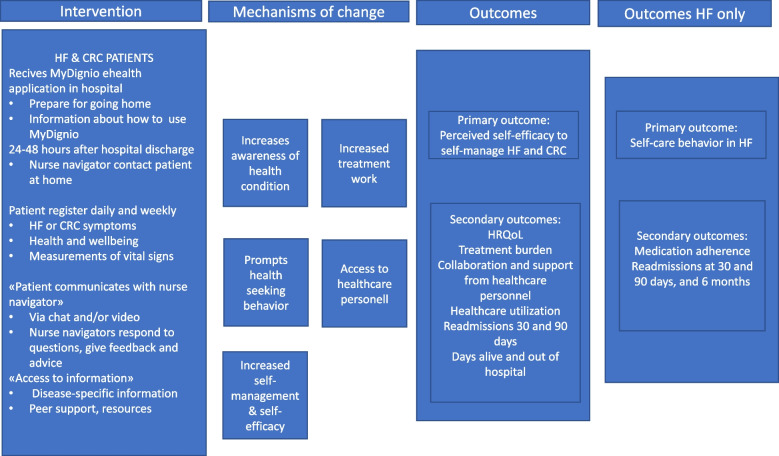
Logic model of the eHealth@Hospital-2-Home intervention

The logic model for the intervention [46] and RCT (see Fig. 1) illustrates the resources needed (i.e., intervention) to implement change (i.e., mechanisms of change) that may lead to the result we intend to achieve (i.e., outcomes).

## Methods

### Study design and setting

**Fig. 2:**
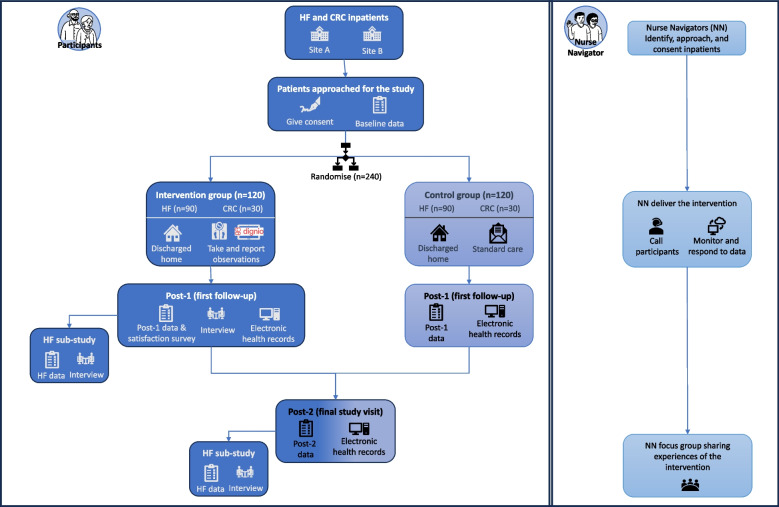
Overview of the RCT trial design

This study is an RCT with an embedded evaluation of intervention engagement and cost-effectiveness. The RCT is part of a larger study ‘Nurse-assisted eHealth service from the hospital to home: Ameliorating the burden of treatment among patients with noncommunicable diseases’ funded by the Norwegian Research Council (Funding No. 301472). An overview of the trial design is shown in Fig. 2.

The trial will include study participants with HF and CRC from two similar-sized university hospitals in Norway (Study Sites A and B). Study Site A is a university hospital in the western part of Norway that provides specialist health care for approximately 370,000 inhabitants. Study Site B is a university hospital in the middle of Norway, providing specialist health care for approximately 300,000 inhabitants (as of September 2023).

### Eligibility of study participants

Eligible patients will be adults aged ≥ 18 years with either HF or CRC chronic illness admitted to the hospital, and able to speak and write in Norwegian. See Table 1 for detailed illness-specific eligibility criteria.

**Table 1:** Eligibility criteria

	**Heart failure**	**Colorectal cancer**
**Inclusion**	• Inpatient with symptomatic HF • Decompensation symptoms such as dyspnoea at rest, pulmonary congestion (X-ray, oedema, or positive rales on auscultation) and elevated NT-proBNP > 1000 pg/mL	Surgically treated for cure stage I–III colon or rectal cancer [47]
**Exclusion**	• Waiting list for a heart transplant • Requires LVAD • Life expectancy < 6 months	• Metastatic cancer • Clavien-Dindo Surgical Complication Score > 3a [48] • Life-threatening acute medical emergencies (e.g., myocardial infarction)
	• Severe mental illness or cognitive impairment • Planned discharge to a nursing home • Participation in other intervention studies • Unable to stand independently	

*HF* Heart failure, *LVAD* Left ventricular device, *NT-proBNP* N-terminal pro b-type natriuretic peptide

### Sample size calculations

The sample size goal is 240 patients (HF = 180, CRC = 60) equally divided between the intervention and control group. Assuming a 20% dropout, a sample size of 240 will provide a power of 80% to detect, at an α error probability of < 0.05, a medium-sized standardised difference in the primary outcome (self-efficacy for managing chronic disease) of 0.4 in the disease groups combined at the first time point (Post-1). For the HF sub-study, assuming the same dropout rate of 20%, there will be 80% power to detect a standardised effect of size 0.5 for the outcome of self-reported self-care.

### Patient screening and recruitment

All patients will be screened and recruited during their hospital admission. Patients with HF will be recruited from both sites, whereas those with CRC will be recruited from a surgical ward at Study Site A. Hospital ward patient lists will be screened by the nurse navigators daily for eligible patients using the eligibility criteria (see Table 1). Nurse navigators will approach those who are eligible, providing written and oral study information. A short, animated video about the research project and the nurse-assisted eHealth intervention will be provided for viewing [49]. The patient will be included if informed written consent has been provided.

### Randomisation

Patients who agree to participate will be randomised. Randomisation occurs after the patients have completed the baseline assessment. Participants will be allocated by stratified block randomisation (1:1) to either the intervention or control group using sequentially numbered, opaque, sealed envelopes. Stratification will occur by diagnostic groups (HF and CRC) and study sites. Separate randomisation lists will be produced for all three strata by a statistician (ID) and concealed from researchers. Randomisation allocation will be blinded for statisticians and researchers performing the data analyses.

### Intervention group: eHealth@Hospital-2-Home intervention

The eHealth intervention is a non-acute, alert- and communication-based service operated by nurse navigators. The intervention duration is six weeks following hospital discharge and aims to monitor and support participants.

#### Participants

Participants in this group will be issued an iPad with only the eHealth application MyDignio. MyDignio uses a cloud-based secure database that can be accessed without an IP address, allowing information exchange using an encrypted message exchange system. Communication via chat and video links between the patients and the nurse navigators in the hospital is supported in the application. MyDignio app is linked to monitoring devices by Bluetooth for each participant to record clinical measures (i.e., blood pressure, pulse, temperature and weight).

Participants will be asked to register clinical measurements HF and CRC daily, register symptoms daily and weekly, and register well-being daily (see Table 2 for overview) into MyDignio app, which will be monitored by nurse navigators employed at the two hospitals [41]. Participants are asked to perform the clinical measurements at approximately the same time to minimise daily variance caused by meals, fluid intake and bowel movements. They will receive a reminder in the system if they have not reported the data. Additionally, participants can communicate with the nurse navigator at any time via chat and video links through the application. Before discharge from the hospital, participants will receive training from the nurse navigators on the use of MyDignio app and the monitoring devices. Instructions and contact details for accessing technical support will be provided.

**Table 2:** Clinical measurements, symptoms and well-being reported by participants

	**Heart failure**	**Colorectal cancer**
**Daily clinical measurements**		
Body weight	x	x
Temperature 10 days		x
Blood pressure and pulse	x	
**Daily self-reports**		
Well-being	x	x
Side effects of medications	x	
Shortness of breath	x	
Heart failure symptoms	x	
Dizziness and oedema	x	
Nausea, diarrhoea		x
Bowel function		x
Redness and pain in surgical wound		x
**Weekly self-reports**		
Perceived side effects of medications	x	
Appetite	x	x
Fluid intake		x
Energy and activity level	x	x
Influence of disease on everyday life	x	
Sleep	x	x
Anxiety and negative thoughts		x
Sadness and loneliness	x	x

#### Nurse navigators

Eight registered nurses will be recruited as nurse navigators to work either with HF (nurse navigators = 4) or CRC (nurse navigators = 4). The nurse navigators are experienced hospital nurses with the clinical skills required to identify and monitor high-needs patients. Digital and in-person training (13 h) is provided in the study procedures and using DignioPrevent.

DignioPrevent is the clinical interface to the eHealth intervention application. Nurse navigators use DignioPrevent to monitor and respond to participant data. They will contact participants 24 to 48 h after their hospital discharge to offer further support in using the MyDignio app. Nurse navigators also monitor participants using DignioPrevent to respond to messages daily and schedule calls. The private messaging system in DignioPrevent allows nurse navigators to answer questions about the patient’s health and provide feedback and advice to the participants about medications, nutrition, fluid intake and activity based on the participant-reported data, facilitating informed and supportive self-care. Nurse navigators can also provide information about available websites with information about HF and CRC, resources and peer support services relevant to the patient’s situation.

Each participant profile will have personalised predefined thresholds that trigger green, yellow or red flags. Yellow and red flags require the nurse navigator to respond to those patients within 48 h. Red flags require the nurse navigator to advise the patient to first conduct extra clinical measures to ensure that they are correct; and second, advise the patient to contact their general practitioner or the municipal emergency department to ensure proper treatment, as the intervention is not an emergency service. Nurse navigators can consult with the medical doctor or surgeon collaborating with the research project to provide medical oversight for the trial about possible medical interventions. The intervention group is considered low risk to participants and provides more support than routine care. Actions to mitigate participant risk include setting specific clinical thresholds on all patient-inputted data, providing research-specific training and provisioning technical support.

### Control group: routine care

The control group will only receive routine care. Routine care refers to the standard care offered after hospital discharge. For all participants in this group, this includes written discharge information, information on how to schedule a follow-up appointment at the hospital outpatient clinic or with their general practitioner if needed, and information to contact their general practitioner or municipal emergency department if worrying symptoms occur after hospital discharge. The participants will receive pertinent disease-specific information as part of routine care for both patient groups. To support participants in this group, they will be contacted by telephone between Post-1 and Post-2 to confirm their continuous participation in the trial and to offer any additional support as required.

### Patient and public involvement

The eHealth intervention was developed in collaboration with representatives from both patient groups and nurses. The representatives aided in tailoring the content and follow-up plan for both HF and CRC patients’ specific health needs post-hospital discharge. Patient representatives and nurses have participated in workshops with the research team to review and assess all information provided to participants as well as the content in the eHealth application MyDignio.

### Outcome measures

An overview of all outcome measures is presented in Table 3.

**Table 3:** Outcomes, measurements and number of items

Outcomes	Measurements	Number of items
**Primary outcome**		
Perceived self-efficacy to self-manage HF and CRC	Self-Efficacy for Managing Chronic Disease [50]	6
**Secondary outcomes**		
Treatment burden	Patient Experience with Treatment and Self-Management Scale [51, 52]	24
HRQoL	EuroQol EQ-5D-5L questionnaire [25]	5
Collaboration with healthcare services	CollaboRATE [53, 54]	3
Support from health professionals	Constructive support from health professionals [55, 56]	12
Healthcare utilisation	Patient self-reports of number of visits to the primary healthcare service and/or the specialist healthcare service	
Readmissions at 30 and 90 days	Count of readmission from electronic medical records	
Days alive and out of the hospital [57]	Patient self-reports of number of days alive and out of hospital	
**HF sub-study outcomes**		
Self-care behaviour in HF	European Heart Failure Self-Care Behaviour Scale [58]	9
Readmissions at 30 and 90 days and 6 months	Count of readmission from electronic medical records	
Medication adherence	Medication Adherence Reasons Scale-5 [59]	5

*CRC* Colorectal cancer, *EuroQol EQ-5D-5L* EuroQoL 5-Dimension 5-Level, *HF* Heart failure, *HRQoL* Health-related quality of life

#### Primary outcome measures

The primary outcome measure for this study will be patients’ self-reports of self-efficacy to manage their HF or CRC disease as measured by the six-item questionnaire Self-Efficacy for Managing Chronic Disease developed by Lorig et al. [50]. The items ask participants to rate their confidence in doing activities across several domains common for many chronic diseases (i.e., symptom control, role function, emotional functioning and communicating with physicians). All items are scored on a 10-point scale (1 = not at all confident to 10 = confident). The total score is the mean score of the six items.

#### Secondary outcome measures

Multiple measures will be used to examine secondary outcomes. These are outlined in Table 3 and briefly described below:

- Treatment burden will be measured with the Norwegian Patient Experiences with Treatment and Self-Management [51, 52]. The four dimensions—medical information (seven items), monitoring health (four items), medications (seven items) and medical appointments (six items)—will be used to focus on treatment burden related to areas of non-adherence in chronic illness.
- HRQoL will be measured using the validated and reliable EuroQoL 5-Dimension 5-Level (EQ-5D-5L) questionnaire [25]. It includes five health dimensions: mobility, self-care, usual activities, pain/discomfort, and anxiety/depression. Patients are asked to rate their current health status with responses that range from no problem to an extreme problem. It includes the EuroQoL visual analogue scale, which is a one-item visual analogue scale where patients report on ‘your health today’ using a scale between 0 and 100.
- Perceived support from healthcare personnel will be measured using 12 items on constructive support [55, 56]. All items are measured on a five-point Likert scale, ranging from ‘agree strongly’ to ‘disagree strongly’.
- Collaboration with healthcare personnel will be measured using the validated three-item questionnaire CollaboRATE [53, 54]. The three items represent three core shared decision-making activities: explanation of the health issue, preference elicitation and preference integration [60]. Each item is scored on a 10-point anchor scale from 0 (‘No effort was made’) to 9 (‘Every effort was made’).
- Healthcare utilisation will be captured using patients’ self-reporting of the number of visits to the primary healthcare service (i.e., general practitioner, municipal emergency department) and/or the specialist healthcare service (i.e., outpatient clinic).
- Days alive and out of hospital [57] will be measured by comparing a patient’s self-report data with hospital records and subtracting the number of days spent away from home due to HF- or CRC-related hospitalisation from the first reporting in MyDignio up to six months (Post-2) [61].
- Numbers of readmissions within 30 and 90 days will be collected from the electronic healthcare record system in the hospital.

#### HF sub-study outcome measures

Participants with HF will participate in a sub-study that aims to explore the effects of the digital eHealth intervention on HF self-care behaviours, readmissions and medication adherence. The primary outcome of this study uses the revised European Heart Failure Self-Care Behaviour Scale, a validated questionnaire comprising nine items [58], to evaluate any changes in self-care behaviours within and between the groups. Secondary outcomes will examine the number of readmissions at 30 days, 90 days and 6 months from the electronic healthcare record system in the hospital. The validated Medication Adherence Reasons Scale-5, which consists of five items [59], will be used to measure medication adherence and non-adherence behaviours for between-group comparisons.

### Patients’ and nurse navigators’ engagement with the intervention

Engagement [62] with the eHealth intervention will be assessed and explored against self-efficacy for any observable relationship. According to the World Health Organization [63], intervention adherence is the extent to which the patient’s behaviour corresponds to the intervention assigned to them. In this RCT, adherence will refer to the dose received by the participants or the extent to which participants actively engage with, are receptive to, and/or use the materials (i.e., symptom checklists) or recommended resources (i.e., monitoring equipment) [64]. Adherence will refer to engagement, for which adherence is set at 80–100% of the dose delivered (i.e., completing both daily measurements and symptom checklists). Similarly, nurse navigators’ engagement with the digital application will also be explored and evaluated. Finally, engagement data will be used to estimate cost-effectiveness.

### Data collection

Demographic variables collected will include sex, age, education, work, living situation, other health conditions, frequency of contact with the hospital and use of digital tools. Questionnaires will be administered electronically or postally (by participant choice) at three different times: baseline, the end of the intervention (Post-1), and 6 months after baseline (Post-2). The baseline measurements will be collected in the hospital, where Post-1 and Post-2 questionnaires will be completed by the patients at home (electronically or paper, by patient choice).

Interviews will be conducted with participants in the intervention group (*n* = 30, HF *n* = 20, CRC *n* = 10) to explore the experience with the intervention around the themes of usability and acceptability and to inform the results of the intervention effects and future implementation [42]. Participants in the intervention group will also complete the Post Study System Usability Questionnaire [65] to assess satisfaction with the usability of the MyDignio app. Additionally, participants in the HF sub-study (*n* = 12) will be invited to interview twice (Post-1 and Post-2) about their experiences on HF self-care. Interviews with the nurse navigators (*n* = 8) will be conducted in one focus group. Questions will focus on their experiences of delivering the intervention and factors influencing the implementation of the intervention in the hospital. All interview guides will be co-created with patient representatives and the project group. The interviews will be conducted at the location chosen by the patients and nurse navigators. Interviews will be audio recorded, transcribed verbatim and translated into English.

### Data management

Collection of personalised data is limited and only includes personal data necessary for the trial. The Services for Sensitive Data (TSD) administered by the University of Oslo [66] will be used to retrieve, store and process data from the questionnaires and interviews. Electronic questionnaire data will be retrieved using an application that includes an integrated solution for collecting sensitive data (Nettskjema), which is linked to the TSD [66]. All data containing personal identifying information and clinical variables obtained from patient’s medical records will be stored separately on research servers at Sites A and B. Only the project manager and nurse navigators will be able to access the code list connecting personal identifying data to the individual participant.

### Statistical methods

The primary assessment of the intervention effect will be performed using a mixed-model approach to the analysis of covariance (ANCOVA) with the two post-intervention scores of self-efficacy to self-care (at Post-1 and Post-2) as the dependent variable and pre-intervention self-efficacy to self-management, measurement occasion and intervention group as independent variables. The difference in treatment effects between the time points will be determined with an interaction term between the measurement occasion and intervention group. Important predictors of self-efficacy to self-managing HF or CRC or dropout will also be included in the model, as well as the stratifying variables used in the randomisation. Random effects will be included to address dependencies between repeated measures and with dropouts. The sub-study analyses of HF self-care behaviour and medication adherence comprise the primary assessment of intervention effect using the same approach. All analyses will be conducted according to intention-to-treat principles (i.e., including all participants as randomised, disregarding adherence to the assigned treatment). The main comparison will be at the six weeks (Post-1) timepoint. The analysis of secondary continuous outcomes will be performed similarly, whereas readmissions will be analysed using Cox regression. The computer software SPSS [67] and R [68] will be used for analysis and statistical calculations.

### Qualitative data analysis

The text transcripts from the interviews with patients and the focus group interview with nurse navigators will be analysed using thematic analysis [69]. The analysis process will be iterative, in which members of the research team independently read and code the text transcripts. The codes will be sorted into preliminary themes, which will be discussed by the research team before concluding on the final themes to be reported. The software tool NVivo will be used [70].

### Cost-effectiveness

Cost-effectiveness analyses will be performed at both time points, informed by the EQ-5D-5L. Data on resource use in the hospital (inpatient stay, outpatient visits) will be collected from the hospital’s electronic medical records, while use of health- and care services in the municipality (visits to the general practitioner, emergency room and home health care) will be collected from standardised questionnaires in the patient’s home. We will estimate a standard cost for the intervention per patient. The intervention cost includes the digital equipment (e.g., tablet, scale), costs of the nurse navigators’ working hours connected to the patient that receives MyDignio and costs connected to nurse navigators’ weekly work hours spent following up with patients through DignioPrevent.

### Data monitoring and auditing

Data monitoring will be continuously performed by the research team. Nurse navigators will attend regular meetings with the research team to report on their activities and challenges. The researchers have access to the Dignio Prevent system and can remotely monitor nurse navigators’ actions. Twice a year, we will conduct project meetings with a scientific advisory committee (SAC). The SAC members are internationally recognised leading experts in the fields of HF, cancer care, self-care interventions, complex intervention studies, eHealth innovation and health services research. The SAC will provide advice and monitor trial progress.

### Research ethics

The protocol received ethical approval from the Western Regional Committee for Medical and Health Research Ethics (REC west) (REC ID 556114) on 2 February, 2023. Local approvals for each recruiting site were obtained. The research will be conducted according to the Helsinki Declaration [71]. The patients will be included in the study when informed written consent has been provided. The RCT began recruiting in May 2023. Participants will have the right to access the registered information and to correct any errors found in the information. Participants have the right to withdraw from the study at any time. All information will be processed without directly recognisable information. A code will link the participant to the registered information through a list of names that only the project manager can access. The data collected from patients will be stored in a secure research data server (i.e., TSD) until 2028.

De-identified clinical (i.e., hospitalisations) and self-report (self-care behaviour) data on HF patients will be shared with collaborators at Vestre Viken Trust, Drammen Hospital, who are conducting a similar clinical trial (registered in ClinicalTrial.gov #NCT05447598). The purpose of this data sharing is to increase the participants’ contributions to generalisable knowledge, by potentially facilitating additional findings beyond the original, prespecified clinical trial outcomes. The HF participants will be informed about data sharing during the consent process.

### Ancillary and post-trial care

All participants who were surgically treated for CRC will, as part of standard care, attend a post-surgical follow-up appointment at the gastrosurgical outpatient clinic and systematic follow-up according to national guidelines. Patients with HF will be followed up by their general practitioner or at an HF outpatient clinic. All participants will receive a follow-up call when the study is complete with information that the study has ended, how they can access information about trial results and to ask if they have feedback to provide to the research team.

### Dissemination

Trial results will be published as scientific articles in peer-reviewed academic journals. We will also share the results with stakeholders of the project through patient organisation channels, at seminars for relevant healthcare personnel and regularly as popular science contributions in local and social media.

## Discussion

HF and CRC are two major illnesses representing challenges to healthcare systems and self-care for patients. The two illnesses are different in terms of treatment and course of illness [10, 11], but the patients share a similar need for follow-up care to support their self-management after hospital discharge. eHealth interventions and remote follow-up of patients may be a possible solution to patients’ need for support after hospitalisation but will require the development and testing of robust interventions with the two patient groups to ensure that their shared needs are addressed. The intervention presented in this protocol was developed using the Medical Research Council Complex Interventions Framework [42]. It is built on a solid foundation of literature reviews [35, 39] and feasibility research [15, 43] and has been informed by HF and CRC patients, representatives from the two patient groups, and clinicians. Currently, the feasibility data are undergoing analysis, but preliminary results indicate that the intervention meets the shared post-hospital needs of the two patient groups and that an RCT is feasible.

This protocol will facilitate the examination and exploration of the effects of the eHealth @ Hospital-2-Home intervention on two prevalent patient groups, HF and CRC. The inclusion of both groups will allow the exploration of a generic framework for an eHealth intervention after hospital discharge, which if effective, could be adapted to other patient groups, upscaled, and implemented into clinical practice. The use of mixed methods will facilitate the exploration of the effects and experiences of patients and nurse navigators, enabling a more in-depth description of the intervention and its effects. It will also enable the refinement of the intervention for future research.

## Data Availability

Not applicable. No data are available.

## Abbreviations

RCT: Randomised Controlled Trial
CRC: Colorectal Cancer
HF: Heart Failure
HRQoL: Health-Related Quality of Life
EQ-5D-5L: EuroQoL 5-Dimension 5-Level
TSD: The Services for Sensitive Data
SAC: Scientific Advisory Committee
